# Perioperative neurocognitive disorders in older patients: a narrative review of current knowledge in 2026

**DOI:** 10.1186/s13741-026-00716-y

**Published:** 2026-07-04

**Authors:** Xingbo Zhou, Jiaxin Yang, Zhiyin Tang, Sheng Yang, Hai Zhao, Fan Yang

**Affiliations:** 1 Shengjing Hospital of China Medical University, San Hao Street 36 Hao, Shenyang, 110004 China; 2 Shenyang Medical College, Huanghe North Street 146 Hao, Shenyang, 110034 China

**Keywords:** Perioperative neurocognitive disorders, Postoperative delirium, Elderly patients, Neuroinflammation, Biomarkers, Delirium, Cognitive dysfunction, Postoperative complications, Neuropsychological tests, Perioperative assessment and management

## Abstract

Perioperative neurocognitive disorders (PNDs) are frequent and severe complications in older surgical patients, encompassing postoperative delirium, delayed neurocognitive recovery, postoperative neurocognitive disorder, and long-term cognitive impairment. These complications lead to prolonged hospital stay, elevated medical expenditure, and compromised long-term quality of life. In this 2026 narrative review, we systematically outline up-to-date evidence on the pathophysiology, risk factors, screening approaches, and evidence-based interventions for PNDs. The core mechanisms involve neuroinflammation, gut microbiota dysbiosis, blood–brain barrier disruption, cerebral hypoperfusion, oxidative stress, and tau hyperphosphorylation. Key risk factors include advanced age, preoperative cognitive impairment or frailty, intraoperative hypotension, deep anesthesia, hypothermia, cardiopulmonary bypass, and suboptimal postoperative pain and sleep control. Bedside tools (Mini-Cog, MoCA, MMSE, FRAIL scale) permit feasible risk stratification; tau-PT217, NfL, S100A12, and GFAP are emerging predictive biomarkers. Dexmedetomidine is a pharmacologic agent that has been extensively studied and has relatively strong supporting evidence. Non-pharmacological interventions and multidisciplinary care are recommended as first-line strategies. Outstanding issues include optimal intraoperative hemodynamic and anesthetic thresholds, causal links between delirium and long-term cognitive decline, and clinical validation of biomarkers. Future research demands large-scale multicenter randomized controlled trials and standardized workflows to strengthen personalized perioperative brain protection in elderly surgical patients.

## Introduction

Cognitive disorders refer to abnormalities in high-level brain processes associated with learning and memory, and with pathological changes such as aphasia, apraxia, agnosia, and ataxia. Defined in 2018, PNDs encompass preoperative cognitive decline, postoperative delirium (POD), delayed neurocognitive recovery (≤ 30 days), postoperative neurocognitive disorder (≤ 12 months), and long-term cognitive impairment (> 1 year) (Evered et al. 2018). POD is an acute subtype of PND, characterized by acute onset of consciousness disturbance within 7 days after surgery. A temporal relationship has been identified between early POD and PND among older patients, as 80% of surgical patients have varying degrees of cognitive dysfunction on the first day after surgery, with a small proportion recovering on the second day postoperatively (Youngblom et al. 2014).

About 47.5% of patients experience delayed neurocognitive recovery after cardiac surgery and 28.9% experience postoperative cognitive impairment (Linassi et al. 2022). Notably, a retrospective study of preoperative cognitive impairment (CI) and POD in a large cohort of elderly surgical patients (CIPOD) found that 20% of patients aged > 70 y had preoperative CI, leading to a higher likelihood of POD and major negative consequences (Weiss et al. 2023). Furthermore, 50% of patients undergoing major surgery are more prone to POD, which leads to adverse outcomes such as longer hospital stays, increased healthcare expenses, and higher rates of re-hospitalisation (Hughes et al. 2020). Among them, the confusion assessment method (CAM) is often used to evaluate POD, which is found to be more time-saving and effective. The incidence of concurrent POD after mixed (non-cardiac) surgery, orthopedic surgery, and oncology surgery assessed using CAM was estimated at 23%, 27%, and 19%, respectively (Ho et al. 2021). Furthermore, the modified frailty index(mFI) predicts POD and delayed neurocognitive recovery after total joint replacement and can be used to predict risk stratification before surgery (Chen and Qin 2021). PND is currently diagnosed through cognitive function testing.

The pathogenesis of PNDs involves multifactorial interactions between patient-specific vulnerabilities and perioperative insults. Advanced age, preexisting neurodegenerative changes, metabolic dysfunction, and frailty are key predisposing factors. Surgical trauma and anesthesia-related neurotoxicity further amplify these risks. Neuroinflammation is a key research focus of PND pathogenesis: Surgical stress triggers peripheral release of pro-inflammatory cytokines (IL-1β, IL-6, TNF-α), activating microglia and disrupting neuronal function (Liu and Zhang 2025). Preoperative corticosteroid administration can attenuate surgical inflammatory response and thereby reduce the incidence and severity of early and late PND, as confirmed by two classic studies (Glumac et al. 2017, 2021), highlighting inflammation as a key modifiable target. Emerging evidence highlights the critical role of gut microbiota dysbiosis in modulating systemic inflammation and contributing to neuronal injury (Wei et al. 2024).

Current management strategies emphasize the adoption of multimodal approaches. Pharmacologically, anti-inflammatory medications, such as dexmedetomidine have shown beneficial effects in mitigating neuroinflammation and reducing the severity of delirium (Cui et al. 2020). Non-pharmacological interventions, such as early postoperative mobilization, play a critical role in reducing risks (Feng et al. 2024).

**Fig. 1:**
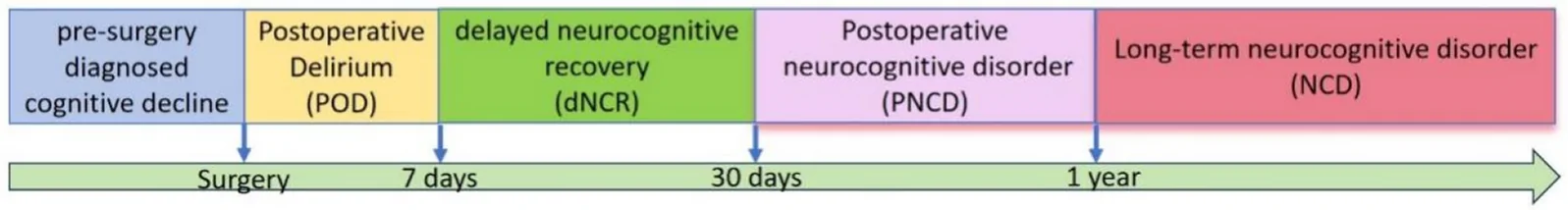
Postoperative neurocognitive disorders (PND)

PNDs profoundly impact both perioperative outcomes and long-term brain health, underscoring the need for early risk stratification and personalized interventions. This article reviews the latest advances in basic and clinical research regarding the mechanisms, pathophysiology, influencing factors, and treatments of postoperative neurocognitive disorders (PNDs), identifies established risk factors and effective interventions, and points out unresolved scientific issues and future research directions, so as to provide references for early clinical intervention and postoperative management (Table 1) (Fig. 1).

**Table 1:**
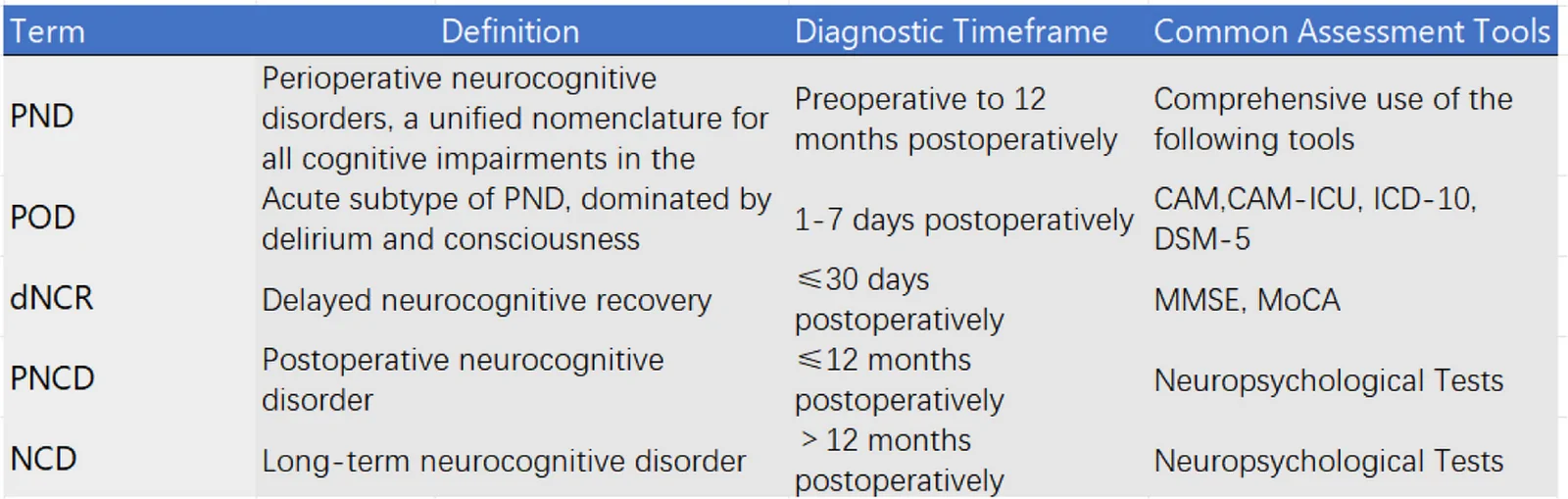
Definitions, diagnostic timeframes, assessment tools of PND and subtypes

## Methods

This narrative review synthesizes current evidence on perioperative neurocognitive disorders (PNDs) in older surgical patients. A literature search of PubMed, Scopus, and Web of Science was conducted in March 2025, focusing on English-language, human studies published between January 2020 and March 2025. Keywords included “perioperative neurocognitive disorder,” “postoperative delirium,” “elderly,” and “surgery.” Titles and abstracts were screened for relevance, and full texts of selected articles were reviewed. Information was organized thematically (e.g., pathophysiology, risk factors, management), and findings were critically evaluated to distinguish established knowledge from areas of uncertainty. The review adheres to the 2018 PND nomenclature.

## Pathophysiology of perioperative cognitive dysfunction

**Fig. 2:**
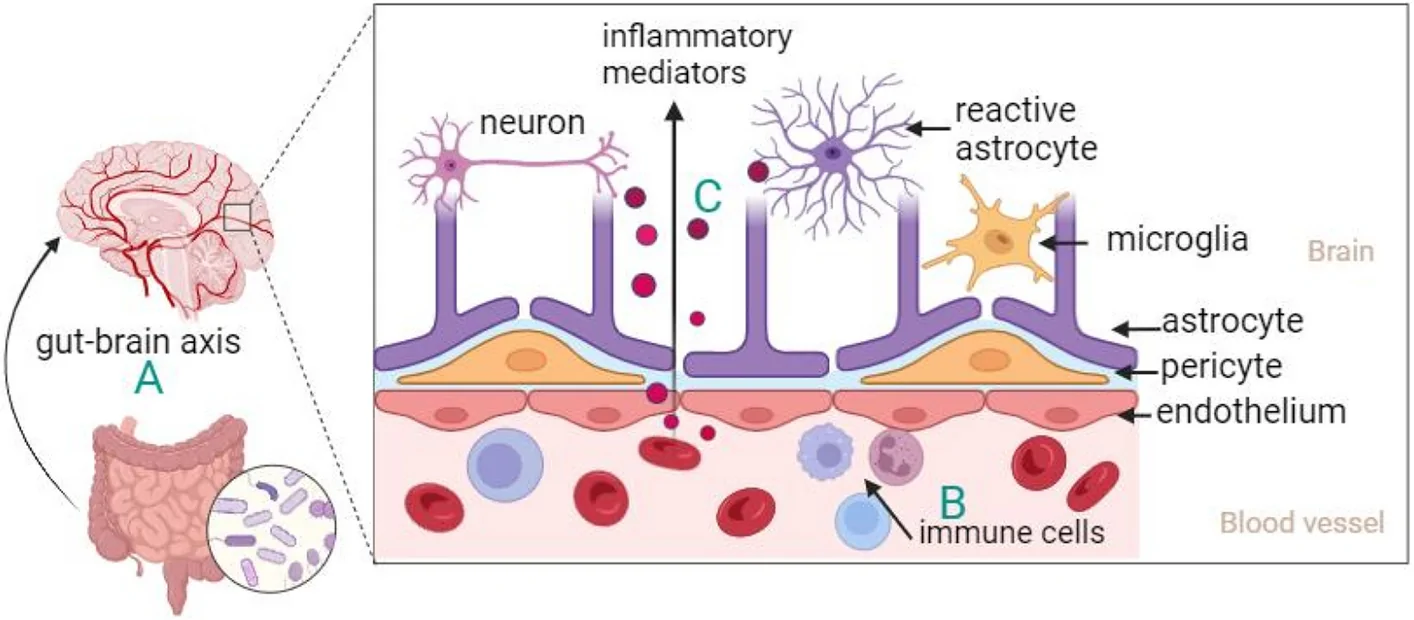
Mechanisms of PND. **A** Gut microbes can communicate with the central nervous system through neural, immune and endocrine pathways (gut-brain axis) and affect brain function. A gut microbial imbalance can damage the intestinal barrier, cause metabolic abnormalities, and lead to neuroinflammation. **B** The immune vascular hypothesis states that innate immune disorders might adversely affect the blood–brain barrier and cause POD in patients. **C** Surgery under anaesthesia can induce neuroinflammation and activate astrocytes and microglia, thus causing neuronal damage

Perioperative cognitive dysfunction might be associated with neuroinflammation, neuronal damage and intestinal microbial dysbiosis. For example, tau protein with threonine 217 (tau-PT217) and neurofilament light protein (NfL) might serve as biomarkers of postoperative delirium (Lu et al. 2023; Liang et al. 2023; Reekes et al. 2023; Leung et al. 2023; Fong et al. 2020). Reduced levels of serum brain-derived neurotrophic factor (BDNF), increase nucleotide-binding domain, leucine-rich–containing family, pyrin domain–containing-3 (NLRP3) inflammasomes, endothelial nitric oxide synthase (eNOS), dysfunction and increased plasma interleukin factors are all associated with the occurrence of PND in elderly patient (Travica et al. 2022; Li et al. 2022; Wang et al. 2023; Danielson et al. 2020; Katusic et al. 2023; Hirsch et al. 2016; Brattinga et al. 2022). High mobility group box 1 (HMGB1), a ubiquitous nonhistone nucleosomal protein, may activate circulating bone marrow-derived macrophages and promote their migration to the brain, a process associated with postoperative cognitive decline in preclinical and small-sample clinical studies (Vacas et al. 2014). Relationships between immune factors and surgically-induced neuroinflammation and cognitive dysfunction are becoming clearer. Innate immune dysregulation adversely affects the blood–brain barrier and can cause neuroimmune and vascular damage (Wang et al. 2020). It may predict the risk of patients developing delirium and delayed neurocognitive recovery. In addition to immune dysregulation, cerebral perfusion disturbance and oxidative stress are also important pathophysiological factors of PNDs: reduced cerebral blood flow leads to neuronal hypoxia and injury, while excessive reactive oxygen species disrupt mitochondrial function and accelerate neuronal apoptosis. The balance of gut microbiota significantly influences perioperative cognitive disorders and may contribute to neuroinflammatory responses Preoperative gut dysbiosis elevates the risk of PND in elderly patients and is associated with increased serum levels of pro-inflammatory cytokines (e.g., TNF-α, IL-1β) and metabolites such as valeric acid and palmitamide (Wei et al. 2024). Thus, perioperative cognitive dysfunction might be minimised and neuroinflammation might be reduced in the future by regulating the immune response, and adjusting the intestinal flora of patients. However, the pathophysiological mechanisms of PND remain incompletely elucidated and are predominantly multifactorial (Fig. 2).

## Analysis of risk factors for perioperative cognitive disorders

### Preoperative factors

The risk factors for PND in elderly patients comprise preoperative cognitive function and general health status, intraoperative blood pressure, anesthesia depth, oxygen and CO_2_ pressure, cerebral oxygen, blood glucose management, intraoperative body temperature, fluids, and cardiac surgery. Postoperative factors comprise nursing quality and pain management. However, methods of inducing and maintaining anesthesia are currently believed not to significantly impact POCD, and the effects of anaesthetics remain controversial.

#### Age

Cognitive impairment is associated with advancing age, comorbidities, and lower incomes (Daelman et al. 2024). Aging and baseline cognitive impairment, are consistently related to PND (Evered and Silbert 2018). Aging also causes physical changes, such as reduced immunity, weakness, and so on and high-risk surgeries impose increased risk of persistent functional and cognitive decline (Suwanabol et al. 2022).

#### Preoperative cognitive decline

Complications of total hip arthroplasty that are associated with extant CI[31]comprise increased POD risk, hospital mortality, and length of stay (LOS) (Viramontes et al. 2019). A relationship between baseline CI in older patients undergoing surgery spinal deformity and POD suggests that preoperative CI is a potential risk factor for POD, potentially as a result of reduced preoperative cognitive reserve. Preoperative assessment of CI is recommended for elderly surgical patients at high risk of PND (e.g., hip fracture, cardiac surgery), which helps identify individuals in need of personalized cognitive protection strategies (Paternò et al. 2026; Peden et al. 2021). Preoperative ketamine-associated intraoperative electroencephalography (EEG) characteristics have revealed ketamine-related EEG changes in a heterogeneous group of older patients undergoing spinal surgery; patients with baseline CI who are treated with ketamine are more prone to POD (Barreto Chang et al. 2022). Preoperative CI in older patients increases the risk of POD, LOS, and admission to intensive care units after cardiac surgery (Au et al. 2023).

#### Sleep disorders, anxiety, and depression

Perioperative sleep disorders could increase the likelihood of postoperative neurocognitive disorders, and improving perioperative sleep quality might lower the incidence of PND (Wang et al. 2021). Perioperative sleep fragmentation (SF) and surgery independently cause notable memory impairment; perioperative SF also increases hippocampal inflammation that does not result in CI (Vacas et al. 2017). Evidence for the role of obstructive sleep apnoea in postoperative neurocognitive disorders is limited and requires perioperative investigation (Devinney et al. 2022). Preoperative anxiety in older patients undergoing elective orthopaedic surgery predicts risk of POD. Hence, reducing preoperative anxiety might help to prevent POD (Ren et al. 2021). Anxiety and depression also increase risk of POD and PND among elderly patients (Srifuengfung et al. 2023).

#### Metabolic syndrome and body mass index (BMI)

Patients with metabolic syndrome are at increased risk of POD. Lower levels of high-density lipoprotein cholesterol (HDL-C) are closely associated with POD, whereas an elevated BMI before surgery contributes to PND (Feinkohl et al. 2023).

#### Frailty

Frailty and POD are correlated in older patients undergoing non-cardiac surgery. The occurrence of POD was increased among patients who were deemed frail according to the Fatigue, Resistance, Ambulation, Illness, and Loss of weight (FRAIL) scale, whereas that of PND was not significantly altered (Mahanna-Gabrielli et al. 2020). Frailty is linked to increased 30-day and 6-month mortality rates, postoperative complications and a higher POD incidence (Gong et al. 2023).

### Intraoperative factors

#### Intraoperative blood pressure fluctuations

The association between intraoperative hypotension and the incidence of POD remains debatable. Some studies suggest that intraoperative hypotension is associated with the occurrence of POD. Hidden strokes in the brain caused during surgeries that are unrelated to the heart in terms of low blood pressure, are linked to POD, declined cognitive function, and cerebrovascular incidents one year postoperatively (Marcucci et al. 2023). The results of a randomised control trial (RCT) to determine the effects of various blood pressure control methods on POD in elderly patients who underwent hip replacement discovered that maintaining intraoperative blood pressure > 10% higher than baseline decreased the occurrence of POD and the development of agitation (Xu et al. 2020). Low blood pressure during and after surgery has been linked to the development of POD among critically ill patients under intensive care. Nevertheless, the degree to which these connections are causative remain uncertain (Maheshwari et al. 2020). During cardiopulmonary bypass surgery, Maintaining MAP at > 70, rather than 50–70 mmHg reduces the risk of postoperative neurocognitive decline (Linassi et al. 2022). The POD incidence in older patients undergoing thoracic and orthopaedic surgery was increased when the intraoperative mean arterial pressure (MAP) remained at ≤ 65 mmHg for more than 5 min (Duan et al. 2023). A meta-analysis showed a statistically significant association between IOH and an increased risk of postoperative complications (POD) (Yin et al. 2025). However, other studies have found no association between intraoperative hypotension and the occurrence of POD. A retrospective cohort analysis and An RCT indicates that intraoperative hypotension is not associated with postoperative delirium in elderly patients undergoing elective non-cardiac surgery (Cui et al. 2026). An RCT involving older patients who underwent non-cardiac surgery with general anaesthesia found no link between intraoperative low blood pressure and the development of PND (Langer et al. 2019). In adult patients undergoing elective craniotomy for tumor resection, there was no significant association between intraoperative hypotension and POD (Cui et al. 2026). A recent retrospective cohort study of 38,940 adult patients undergoing non-cardiac surgery reported an overall postoperative delirium rate of 6.56%. No significant association was observed between intraoperative hypotension (defined as the area under the MAP curve below 65 mmHg) or intraoperative mean MAP and postoperative delirium. Although higher time-weighted mean MAP in the postoperative period was statistically associated with delirium, this finding was not clinically meaningful (Rössler et al. 2025). Among 108 patients aged 65–85 years undergoing laparoscopic gastrointestinal surgery, the incidence of postoperative delirium was comparable between patients managed with an intraoperative mean arterial pressure (MAP) target of 65–85 mmHg (group L) and 86–100 mmHg (group H). However, delirium severity was significantly higher in group L which indicates that a higher intraoperative MAP target does not reduce the incidence of postoperative delirium but may mitigate its severity (Zhang et al. 2022). In conclusion, the duration of intraoperative hypotension may be associated with the occurrence of POD, though the optimal mean arterial pressure (MAP) threshold remains controversial.

#### Depth of anaesthesia

Intraoperative depth of anaesthesia is a controversial factor associated with perioperative neurocognitive disorders in older patients. A clinical randomised controlled trial to determine the relationship between anaesthesia depth and delirium found that the incidences of POD were 19% and 28% among patients with a bispectral index (BIS) of 50 and 35, respectively (*P* = 0.010). After 12 months, the incidence of cognitive dysfunction was markedly reduced in the BIS 50 group compared with the BIS 35 group (*P* < 0.001), suggesting that maintaining a moderate light anaesthesia depth with a BIS score of 50 may reduce the risk of POD in older patient (Evered et al. 2021). Moreover, light anaesthesia rather than deep anaesthesia, is associated with a lower incidence of POD and might promote postoperative neurocognitive improvement (Li and Zhang 2020). Notably, the association between anaesthesia depth and perioperative neurocognitive outcomes remains debatable due to heterogeneous study results. A systematic review has failed to identify a significant correlation between intraoperative anaesthesia depth and long-term postoperative cognitive abilities or survival in older surgical patients (Wang et al., 2023). The dose of anaesthetics, the duration under anesthetsia, and characteristics should be considered to reduce POD incidence. Individualised BIS monitoring during general anaesthesia prevents the development of delirium (ED) in children after BIS-guided anaesthesia (Frelich et al. 2024).

#### Intraoperative oxygen concentration and hypocapnia

Low end-tidal CO_2_ (ETCO_2_) under anaesthesia, which is related to intraoperative ventilation management, can predict POD. Lower ETCO_2_ concentrations can cause POD but are not related to the accumulation of anaesthetics (Mutch et al. 2018). Magnetic resonance imaging (MRI) has revealed that cerebral blood flow (CBF) varies with ETCO_2_ and O_2_ levels in conscious adults during anaesthesia and those under critical care. The combined effects of high O_2_ and low CO_2_ concentrations were significant and might lead to postoperative delirium or during intensive care (Mutch et al. 2020). Therefore, maintaining normal gas exchange is crucial and can be achieved through appropriate anaesthesia management, mechanical ventilation adjustments, and monitoring. These measures help to prevent intraoperative hypoxaemia and hypocapnia, and reduce risk of postoperative neurological disturbances and delirium (Ahrens et al. 2023). However, an RCT study found that intraoperative management of different ETCO₂ levels does not change the incidence of postoperative delirium. The impact of intraoperative ETCO₂ on POD remains controversial (Chen et al. 2024). Low end-tidal CO2 (ETCO2) under anesthesia may be a potential predictive factor for POD in some observational studies, but this finding has not been validated in large-sample RCTs. Older patients undergoing cardiac surgery did not experience a decrease in POCD when exposed to normal, as opposed to high O_2_ levels (median partial pressure of oxygen: 153 *vs.* 309 mmHg). This study found that high intraoperative oxygen levels had no significant effect on short-term neurocognitive outcomes after cardiac surgery, but further multicenter RCTs with standardized oxygenation strategies are needed to confirm this conclusion (Shaefi et al. 2021).

#### Optimise cerebral oxygen saturation

Perioperative outcomes can be optimised by intraoperatively managing cerebral tissue oxygen saturation (Sct O_2_). Intraoperative Sct O_2_ has been linked to a decrease in POD occurrence. However, the connection between POCD and Sct O_2_ requires confirmation by several RCTs due to substantial variation among studies (Zorrilla-Vaca et al. 2018). An electroencephalography (EEG) assessment of postoperative brain O_2_ levels and cognitive function among adult patients during non-cardiac surgery found that alpha brainwave power in the back of the head, connections between the front and top of the brain, and monitoring brain O_2_ levels were not linked to postoperative cognitive function (Vlisides et al. 2023). Targeted therapy to optimise cerebral oxygenation can result in better memory outcomes after cardiac surgery (Uysal et al. 2020).

#### EEG

The duration of EEG suppression significantly correlates with frailty and POD among older patients who undergo non-cardiac surgery (Fang et al. 2023). Preoperative cortical thickness is associated with postoperative EEG power and POD severity. These findings might be based on differences in underlying mechanisms or processes (White et al. 2021). The debate over EEG-guided anaesthesia for reducing POD continues. Some argue that the uncertainty of using EEG to decrease POD is due to the absence of evidence supporting a direct connection between anaesthesia depth and POD. Furthermore, the ideal EEG signal mechanism for guiding anaesthetic drug titration is currently unclear and the potential benefits of reducing anaesthesia levels might be offset by risks associated with insufficient anaesthesia (Hao et al. 2023).

#### Fluid management 

Goal-directed fluid therapy (GDFT) helps to maintain stable blood flow in the prone position, enhances organ perfusion and oxygen balance, decreases inflammation, and reduces the occurrence of early POCD among older patients with spinal stenosis (Zhang et al. 2018).

#### Hyperglycaemia

A review of perioperative hyperglycemia and postoperative cognitive outcomes has suggested that diabetes and high blood sugar around the time of surgery might increase the likelihood of developing POD or cognitive dysfunction. However, these conclusions were based mainly on observational findings; the relationship between perioperative hyperglycaemia and postoperative neurocognitive outcomes requires further investigation (Hermanides et al. 2018).

#### Cardiac surgery

Delirium is a common complication after cardiac surgery that is associated with mortality among older patients. Independent risk factors for delirium that frequently develops after cardiac surgery and is associated with mortality comprise low education level, hypertension, mitral valve disease, and atrial fibrillation (Oliveira et al. 2018). Cardiac surgery is a specific high-risk factor for PND mainly due to brain injury induced by cardiopulmonary bypass (CPB): CPB leads to cerebral hypoperfusion and microembolism formation, which damage cerebral microvasculature and neuronal cells; in addition, CPB triggers an excessive systemic inflammatory response and oxidative stress, further amplifying neuroinflammation and neuronal injury (Bruggemans 2013; Hovens et al. 2016; Harten et al. 2012). Systemic inflammatory responses after major cardiac surgery disrupt the executive control network function of the brain including functional connectivity (Zhu et al. 2023).

#### Temperature

Intraoperative or mild hypothermia is associated with an increased incidence of POD (Ju et al. 2023). A Results support a relationship between POD and unplanned perioperative hypothermia (UPH) among adults undergoing noncardiac surgery (Wagner et al. 2021). Intraoperative hypothermia in patients undergoing general anesthesia for gastrointestinal surgery increases the risk of postoperative delirium (POD) (Wang et al. 2024). Intraoperative hypothermia can induce postoperative cognitive dysfunction and delirium through multiple pathways. The core molecular mechanism is that hypothermia inhibits the activity of PP-2A protein phosphatase, leading to hyperphosphorylation of tau protein, which further forms neurofibrillary tangles, damages neuronal structure and causes injury, and directly results in cognitive decline. Meanwhile, hypothermia triggers pathophysiological changes including decreased cerebral perfusion, disordered energy metabolism, activation of inflammation and oxidative stress, as well as ischemic-hypoxic brain injury. In addition, hypothermia mediates delirium through an indirect pathway by inducing postoperative shivering, aggravating incision pain, and disturbing the sleep–wake cycle (Wang et al. 2024; Planel et al. 2007; Whittington et al. 2010).

Active perioperative temperature maintenance (core temperature 36.0–37.5℃) through warm blankets and heated infusion can reduce the incidence of postoperative delirium (POD) in high-risk elderly patients. Studies have demonstrated that perioperative hypothermia is closely associated with an increased risk of postoperative complications, including the development of postoperative delirium (Torossian et al. 2015). Preoperative pre-warming combined with intraoperative warming can maintain body temperature more effectively and improve patients' comfort level (Özsoy and Dolgun 2024). Infusing warmed fluids can significantly increase patients' core body temperature and reduce the incidence of postoperative shivering (Campbell et al. 2015). Through active perioperative temperature management, especially combined with the use of warming blankets and intravenous fluid warming, the core body temperature of high-risk elderly patients can be effectively maintained, thereby significantly reducing the incidence of postoperative delirium. This strategy not only improves patients' comfort, but also lowers the occurrence of postoperative complications, providing an important guarantee for patients' postoperative recovery (Yoo et al. 2021). Research on the association between intraoperative hyperthermia and PND is currently limited, and more studies are needed to confirm its clinical significance.

#### Unrelated or controversial factors

Anaesthesia method: The choice of anaesthesia method appears unrelated to PND severity and complications in patients with or without preoperative cognitive impairment (CI) (O'Brien et al. 2023; Bhushan et al. 2022).

Common anaesthetics such as propofol and sevoflurane may not affect the occurrence of delayed neurocognitive recovery (Li et al. 2021). Some studies imply that early POD may be associated with volatile anaesthetics, particularly in patients aged ≥ 75 years (Saller et al. 2022). In contrast, the amount of administered sevoflurane did not correlate with the severity or frequency of POD in a cohort of older individuals (Taylor et al. 2023). Abrupt cessation of benzodiazepine receptor agonists in the perioperative period may increase POD risk and should be avoided (Omichi et al. 2021). Other studies reported that perioperative benzodiazepine administration did not elevate POD incidence or reduce intraoperative awareness (Wang et al. 2023). Based on limited evidence, cognitive function showed no significant differences between patients sedated with inhaled or intravenous anaesthetics in the ICU (Cuninghame et al. 2023).

### Postoperative factors

#### Quality of care

High-quality perioperative care (orienting communication, oral and nutritional assistance, and early mobilization can reduce the risk of POD (Chen et al. 2017). Interdisciplinary high-quality care provided to high-risk older patients after elective spinal surgery might reduce the incidence of POD, especially among those undergoing major surgery (Pernik et al. 2021). High-quality nursing care exerts a more significant protective effect against postoperative delirium in critically ill patients (Almoliky et al. 2025). Preoperatively, individualized multimodal preventive strategies are implemented for patients, together with geriatric or multidisciplinary consultations, family engagement, and targeted health education. Intraoperatively, monitoring of anesthetic depth and cerebral oxygen saturation is adopted to mitigate the risk of postoperative cognitive impairment. Postoperatively, patients are encouraged to undertake early mobilization, coupled with optimal nutritional support, improved sleep quality, effective pain control, maintenance of fluid and electrolyte balance, shortened indwelling catheter duration, as well as cognitive and sensory support (Yin et al. 2024).

#### Melatonin

Postoperatively elevated melatonin levels might be associated with an increased incidence of POD, but not with POCD. Nevertheless, POD could potentially increase the risk of developing POCD (Mu et al. 2021).

#### Postoperative complications

Elderly surgical patients with cognitive impairment, poor general health, or hip fractures face elevated postoperative delirium (POD) risk. POD raises short- and long-term mortality; frailty, inadequate comprehensive geriatric assessment, and excess perioperative blood loss further worsen outcomes (Bai et al. 2020; Chen et al. 2019; Kim et al. 2013; Deng et al. 2025). Furthermore, individuals who experience postoperative issues such as pneumonia or urinary tract infections are particularly susceptible to POD (Smith et al. 2017). Targeted monitoring, preoperative evaluation, and optimized perioperative care reduce POD risk and improve clinical outcomes in this high-risk group.

#### Postoperative pain

Acute pain in patients after non-cardiac surgery largely mediates the relationship between preoperative CI and delirium. However, the clinical significance of this finding requires further investigation due to a small effect size (Ma et al. 2023). Reduced opioid administration during surgery might increase postoperative pain and opioid consumption. Conversely, reasonable use of intraoperative opioids might improve long-term outcomes (Santa Cruz Mercado et al. 2023). Dexmedetomidine exerts safe sedative, anti-inflammatory, analgesic, and antiemetic effects, offering potential for transitioning opioid-centred pain management strategies to improve POCD (Su et al. 2022).

#### Postoperative clinical characteristics associated with PND/POD

**Fig. 3:**
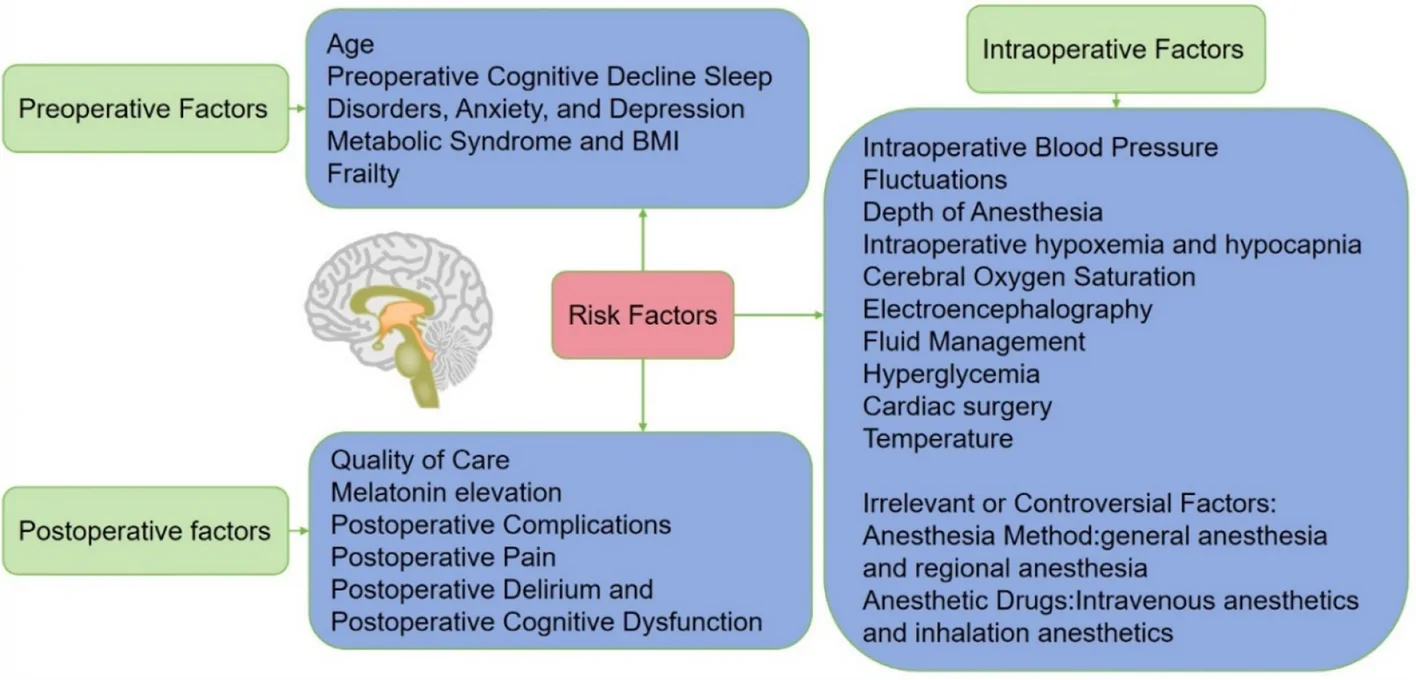
Risk factors for perioperative cognitive disorders

During the first month after surgery, POD significantly increases the risk of POCD, whereas the correlation between them is weak at 2 and 6 months of followup (Daiello et al. 2019). Whether POD is a risk factor for POCD requires further investigation. Others have shown that POD exerts long-term effects on brain networks, leading to a decline in brain connectivity strength at 3 months after surgery, which can significantly impair postoperative cognitive ability (Ditzel et al. 2023). A meta-analysis on the connection between delirium and postoperative cognitive decline found a strong link between delirium and long-term cognitive decline among surgical and non-surgical patients (Goldberg et al. 2020). Another 6-year cohort study found that delirium accelerated the rate of cognitive decline by 40% within 72 months after elective surgery. However, that study was observational (Kunicki et al. 2023). Therefore, whether delirium directly causes subsequent cognitive decline remains unclear. Others have found that POD is an independent risk factor for a decreased QOL at 3 years after cardiac surgery and might lead to cognitive and functional deterioration and increased risk of re-admission (Varga-Martínez et al. 2023). However, more investigation is needed to comprehend the cause-and-effect connection between delirium and cognitive deterioration (Fig. 3) (Table 2).

**Table 2:**
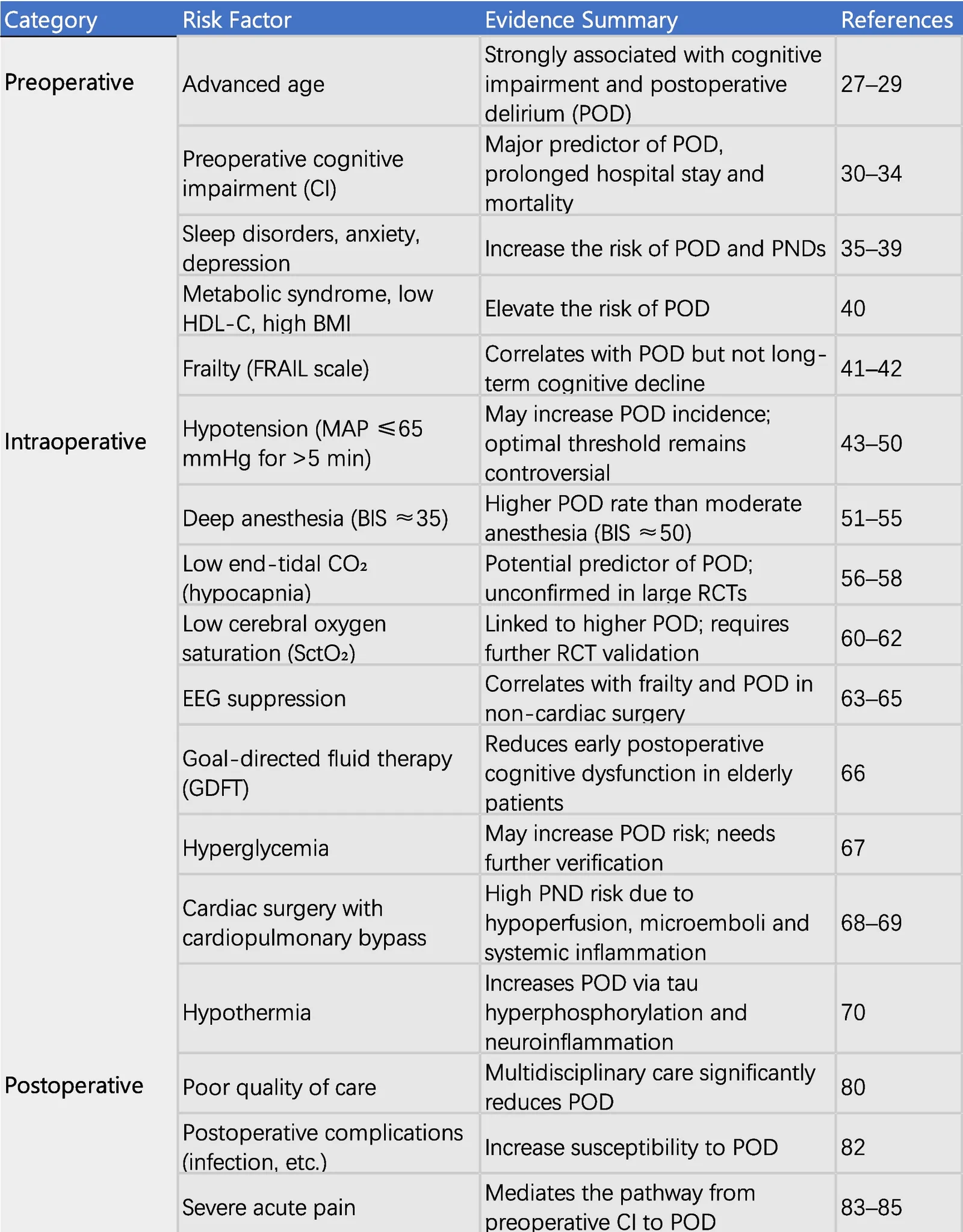
Major risk factors for perioperative neurocognitive disorders (PND)

## Perioperative cognitive disorder diagnosis and screening

This section aims to summarize the validated preoperative screening markers and corresponding clinical tools for PND in older patients, and provide a practical risk stratification scheme for clinical practice.

### Biomarkers

Elevated serum S100A12 levels after hip fracture surgery can lead to inflammation and are linked to both POD and PND independently among older patients. Thus, serum S100A12 could serve as a promising indicator for anticipating POD and PND in older patients (Li et al. 2019). Similarly, the preoperative elevation of t-Tau and p-Tau levels, along with the reduction of Aβ42 levels in cerebrospinal fluid among orthopedic patients, serve as risk factors for the development of POD. Preoperative assessment of cerebrospinal fluid biomarkers can facilitate the prediction of POD occurrence, thereby enabling the implementation of preventive strategies to mitigate the associated risks (Feng et al. 2025). Furthermore, elevated levels of p-Tau-181 prior to surgery may be associated with an increased risk of POD development (Sim et al. 2025). The levels of specific enolase and S100β in the neurons of patients with PND were found to be negatively correlated with the levels of SIRT1 in serum during the early postoperative period. Furthermore, the detection of SIRT1 in serum has been demonstrated to exhibit high sensitivity and specificity. Consequently, the measurement of SIRT1 in serum during the early postoperative phase may serve as a potential biomarker for the clinical prediction of PND (Shi et al. 2024). Multivariable regression has indicated that glial fibrillary acidic protein (GFAP), neuroserpin (NSP) and microRNA-21-5p are the only significant predictors of early PND (ePND). Due to their potential in predicting postoperative delirium and ePND, further investigation into using GFAP, NSP and microRNA-21-5p to monitor postoperative recovery is warranted (Szwed et al. 2021) (Table 3).

**Table 3:**
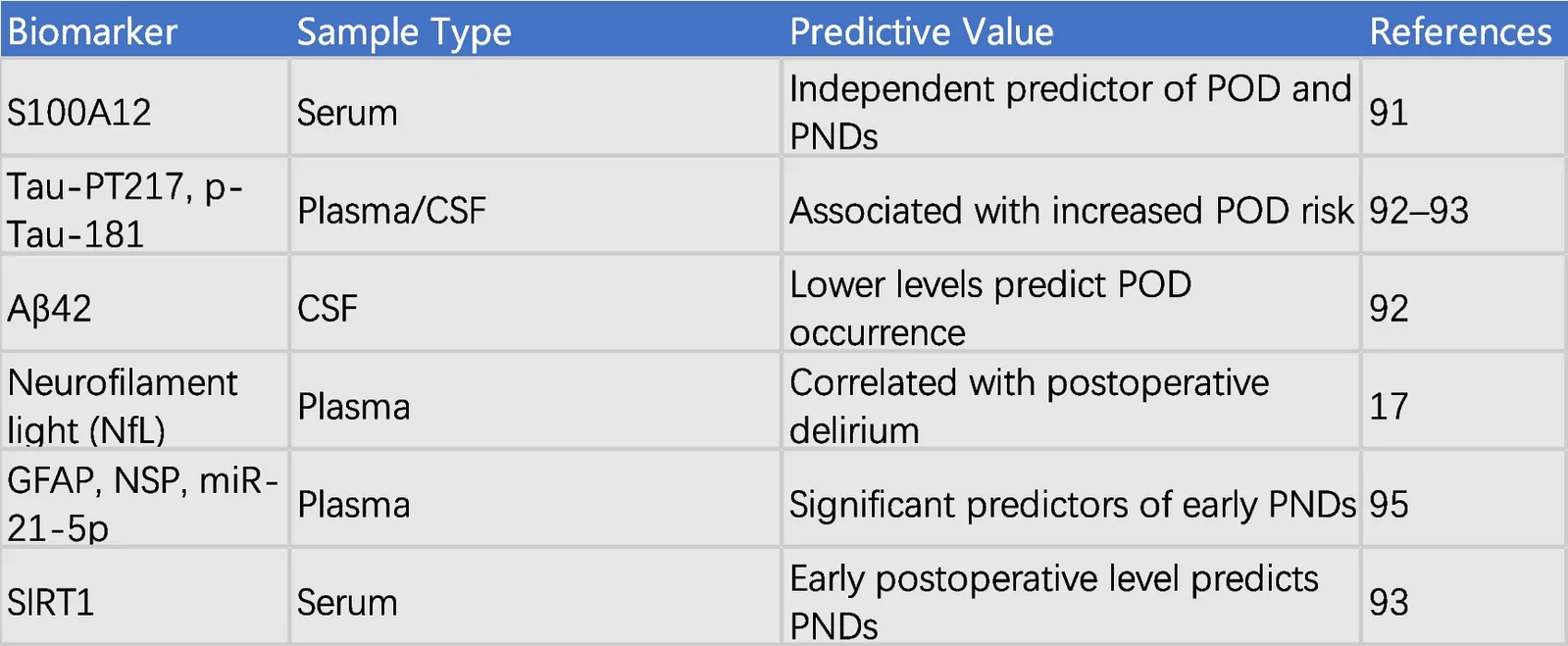
Potential biomarkers for PNDs

### Preoperative cognitive decline

Patients with preoperative CI who are frail and those with only a primary education have lower Mini-Cog scores (Amado et al. 2020). The main screening tools for preoperative CI in elderly surgical patients include the Mini-Cog and Montreal Cognitive Assessment (MoCA): the Mini-Cog is a rapid bedside screening tool a rapid, cost-free screening tool with high sensitivity and specificity for detecting cognitive impairment (CI) in older adults across various healthcare settings, and MoCA is a popular screening measure for mild cognitive impairment (MCI) (Turana et al. 2024; Abayomi et al. 2024; Ratcliffe et al. 2023; Islam et al. 2023). Preoperative CI screening can help identify high-risk patients and implement early cognitive intervention (e.g., cognitive training, optimized anesthesia plan), which is crucial for reducing PND incidence. In addition, the Mini-Mental State Examination (MMSE) is a classic screening tool for moderate to severe CI, with a cut-off value of < 27 for mild cognitive impairment in elderly patients (Arevalo-Rodriguez et al. 2021; Salis et al. 2023).

### Frailty

Frailty is characterised by reduced muscle mass, strength, and nutrition that affect physical performance. Cognitive and psychological functioning is often associated with advanced age (Dammavalam et al. 2023). Preoperative screening and evaluation to identify vulnerable older adults might be a first step towards reducing complications. Frailty indicators are significantly associated with POD among older patients undergoing elective surgery. Therefore, anaesthesiologists can use the frailty index to identify patients at increased risk of POD (Steenblock et al. 2023).

### EEG monitoring

In addition to being routinely used to monitor anesthesia titration, EEG can also serve as a feasible monitoring tool. Preoperative EEG alpha attenuation in patients who are awake and resting with their eyes open might be a neurological biomarker of postoperative attention disorders, and inattention is also one of the characteristics of delirium (Acker et al. 2024). Processed EEG-guided general anaesthesia management using the Patient State Index combined with Density Spectral Array monitoring can significantly reduce the risk of POD in patients undergoing carotid endarterectomy (Xu et al. 2021). Overall, EEG complexity is not associated with delirium or attention. The entropy cross-scale during intraoperative and preoperative periods negatively correlates with changes in delirium severity scores. Therefore, increased average EEG complexity during surgery is related to this scale. The cross-point where preoperative and intraoperative complexities are equal can predict POD remains unclear (Acker et al. 2021). These data do not prove a relationship between processed EEG monitoring and POCD; further investigation is required (MacKenzie et al. 2018). Perioperative EEG can identify brain function integrity as a predictor of POD and POCD among older patients who undergo surgery, rendering it a feasible tool for predicting POD and long-term cognitive decline (Berger et al. 2023). However, more comprehensive investigation is required to establish the role of EEG in predicting POCD. An updated meta-analysis concluded that current evidence is insufficient to support EEG monitoring for the prevention of POD. Therefore, more reliable studies are needed (Sun et al. 2020). The method of anesthesia administration guided by EEG did not reduce the incidence of postoperative delirium among elderly patients undergoing major surgery (Wildes et al. 2019).

### MRI

Confusion and CI after non-cardiac surgeries assessed by MRI found that diminished thalamic and hippocampal size and lower CBF could lead to POCD, whereas changes in white matter pathology before and after surgery could lead to POD. However, the evidence is weak and requires confirmation via large-scale, multicentre, randomised studies. Moreover, future MRI studies are needed to validate the current findings and strive for new breakthroughs (Huang et al. 2018).

### Screening tools and models

#### Bedside screening tools

The FRAIL scale and cognition screening tests (Mini-Cog and Animal Verbal Fluency) for preoperative frailty and CI in older patients can identify populations at high-risk for POD (Susano et al. 2020). Older patients screened for CI using the Mini-Cog on the day of surgery found that test scores related to CI (≤ 2) were closely associated with post-anaesthesia delirium in a care unit (Tiwary et al. 2021).

#### Risk prediction models

The accuracy of multivariable models in predicting POD risk among older patients (*n* = 152) undergoing spinal surgery was 72%, compared with 45% for current electronic medical record-based risk stratification models. That cohort study indicated that age, CI, depression, and surgical complexity are risk factors for POD (Barreto Chang et al. 2023) (Table 4).

**Table 4:**
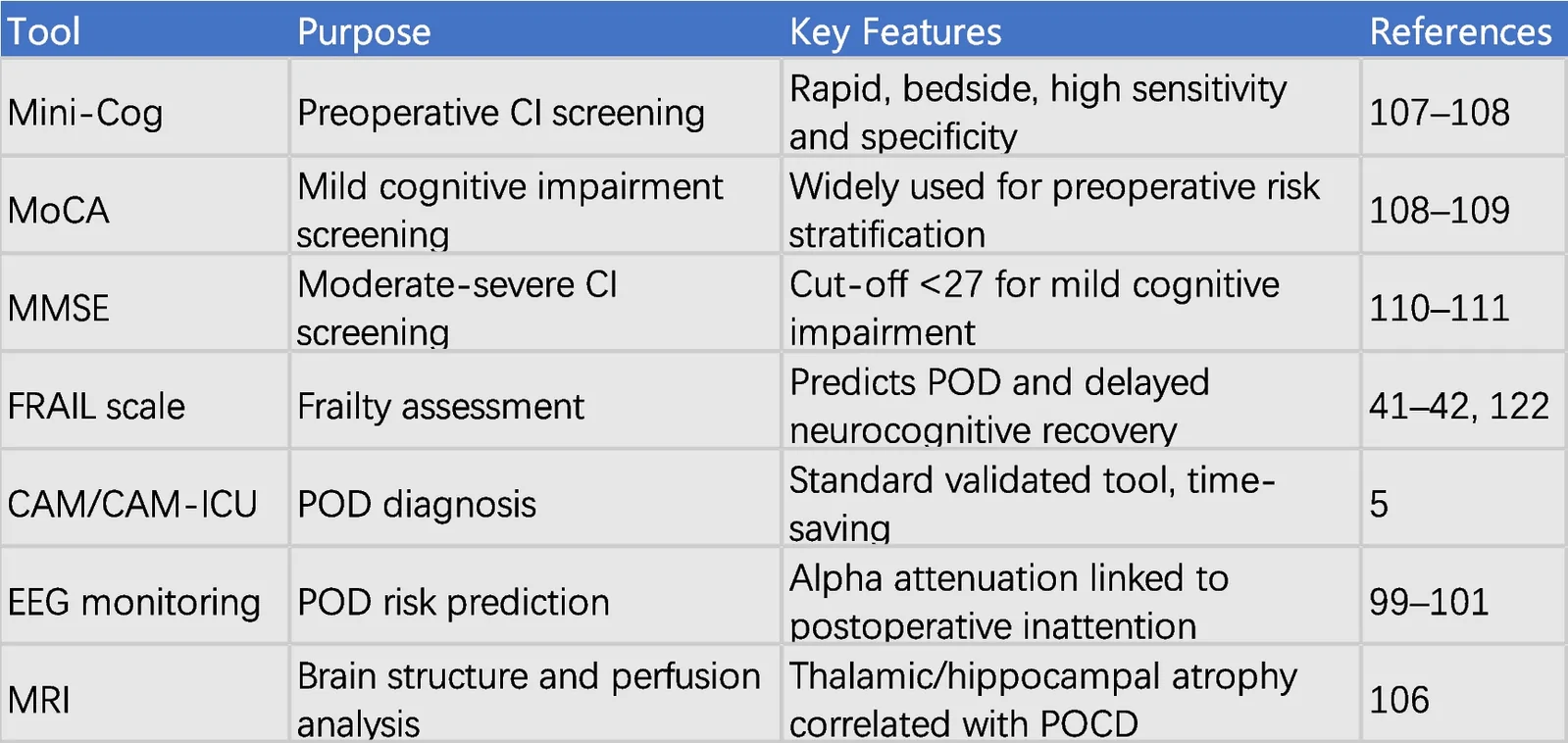
Screening and assessment tools

## Perioperative cognitive disorder treatment

### Medication

Dexmedetomidine exerts anti-inflammatory and neuroprotective properties that can reduce the incidence of acute POD and improve neurocognitive function in PND patients. A continuous dexmedetomidine infusion (0.5 µg kg-1 h-1) has decreased PND incidence within 1 week and improved postoperative sleep, anxiety, and pain in older men undergoing thoracoscopic lobectomy. However, these patients were prone to postoperative bradycardia (Shi et al. 2020). It can lower the rate of acute POD and improve neurocognitive dysfunction in older patients who undergo total knee arthroplasty (TKA). This is unrelated to the regulation of peripheral inflammation by dexmedetomidine, but it is associated with lower plasma S100β concentrations (Mei et al. 2020). Dexmedetomidine reduced the occurrence of PND 1 month postoperatively in older patients undergoing abdominal surgery, possibly due to changes in serum BDNF levels (Cheng et al. 2019). Total intravenous anaesthesia with dexmedetomidine can safely reduce the incidence of POD and the development of agitation in older patients after thoracic oesophagectomy, which is related to reduced levels of the postoperative circulating pro-inflammatory cytokine interleukin (IL)−6 and stable haemodynamics (Hu et al. 2021). In summary, dexmedetomidine is considered an effective sedative for PND/POD prevention (Cui et al. 2020). Dexmedetomidine reduces the incidence of PND after cardiac surgery (Singh et al. 2022). The incidence of POD is more effectively reduced in patients after cardiac surgery when dexmedetomidine is administered postoperatively, than either preoperatively or intraoperatively (Shang et al. 2023). Moreover, dexmedetomidine exerts anti-inflammatory properties by acting on α2-adrenergic receptors, thus inhibiting lipopolysaccharide-induced cognitive decline in mice (Li et al. 2020). A recent meta-analysis has shown that, compared with controls, dexmedetomidine reduced the risk of delayed neurorecovery (DNR) by 41% in elderly patients undergoing noncardiac surgery (Alhamdah et al. 2025). Another meta-analysis demonstrated that perioperative dexmedetomidine significantly alleviated postoperative pain and improved neurocognitive function in patients undergoing orthopedic surgery (Zhang et al. 2025). However, although dexmedetomidine has shown significant efficacy in preventing POD in some studies, several guidelines still do not recommend its use for POD prophylaxis, mainly due to concerns over its cardiovascular side effects and the heterogeneity of treatment effects observed in existing research (Aldecoa et al. 2024).

Perioperative melatonin administration reduced the incidence of delirium among older patients. Although the optimal dose requires further investigation, melatonin and melatonin receptor agonists might become viable options for preventing POD in older patients (Campbell et al. 2019). Edaravone significantly reduces serum chemokine (C-X-C motif) ligand 13** (**CXCL13) and IL-6 concentrations in older patients undergoing hip arthroplasty, thus reducing the incidence of PND (Xie et al. 2021). Cholinesterase blockers such as donepezil (Aricept), galantamine (Razadyne), and rivastigmine (Exelon) can enhance signs of Lewy body dementia and Alzheimer disease by inhibiting the degradation of acetylcholinesterase and decreasing the frequency of disorientation, learning difficulties, and memory confusion. Their adverse effects include dizziness, nausea, and vomiting. Perioperative use of rivastigmine patches can lower the occurrence of POD in older individuals and improve patient outcomes (Youn et al. 2017). Intravenous injections of 2 mg kg-^1^ methylene blue (MB) in older patients undergoing non-cardiac surgery can significantly reduce the occurrence of POD and early PND without a significant rise in perioperative complications, indicating that MB can safely and effectively prevent early postoperative neurocognitive dysfunction (Deng et al. 2021). Preliminary evidence from a single small-sample RCT suggests that probiotics may regulate the gut-brain axis and reduce neuroinflammation, thereby potentially reducing the risk of PND (Gao et al. 2025**).**Probiotics can also be used to prevent perioperative PND development and improve the speech and memory abilities of elderly patients undergoing hip or knee arthroplasty (Hu et al. 2022). However, the efficacy and safety need to be verified by large-sample multicenter RCTs. Neuroinflammatory diseases preferentially impair higher cognitive and executive functions of the prefrontal cortex (PFC). The α2A-adrenergic receptor acts through neural and immune system mechanisms and benefits PFC circuits. Guanfacine acts directly on the dorsolateral prefrontal cortex (dlPFC), reduces stress-related circuit activity and exerts anti-inflammatory effects on the immune system to restore dlPFC network firing and cognitive function, making it a focal point of large-scale clinical trials for treating delirium (Arnsten et al. 2023).

### Non-pharmacological treatment

Non-pharmacological agents are the first-line treatment for PNDs, whereas pharmacological approaches are limited to patients with severe anxiety or distress (Kong et al. 2022). Perioperative Transcutaneous electrical acupoint stimulation can reduce levels of inflammatory IL-6 and tissue necrosis factor (TNF)-α, and lower the incidence of PND among elderly patients undergoing surgery (Wang et al. 2022; Xi et al. 2021). Preoperative psychological preparation plays a crucial role in mitigating postoperative pain, stabilizing psychological changes, and enhancing postoperative recovery. Additionally, interventions aimed at alleviating preoperative emotional fluctuations and cognitive training, such as anxiety, are significant for improving the prognosis of PND and can serve as one of the potential strategies for the prevention and treatment of PND in the future (Powell et al. 2016; Zhao et al. 2023). Exercise including daily activities such as running, swimming and cognitive training also improves cognitive dysfunction-related diseases, including PNDs, thus providing a new strategy for their prevention and treatment (Feng et al. 2024). Moderate exercise stabilises the gut microbiota, reduces neuroinflammation, and improves neuroplasticity as well as cognitive function in aged mice (Lai et al. 2021). Probiotic supplements have a positive impact on cognitive performance and the reduction of inflammatory markers in older adults, the true effect requires further research due to the heterogeneity of RCT studies. The differences in study findings and methodologies reveal the complexity of the relationship between the gut microbiome and cognitive function. Further research is needed to elucidate the potential impact of the gut–brain axis on cognitive health (Coradduzza et al. 2023).

### Multidisciplinary approaches

Implementing multidisciplinary interventions, combining preoperative clinical interventions and medications, and optimising postoperative nursing practices can help to manage POD in older adults (Igwe et al. 2020). Enhanced preoperative collaboration among surgeons, geriatricians, nurses, and anaesthesiologists will help to identify high-risk older patients with complex CI. For example, in elderly patients undergoing cardiac surgery (high PND risk), the multidisciplinary team (MDT) conducts preoperative cognitive/frailty screening, formulates individualized anesthesia/CPB plans, and provides postoperative cognitive rehabilitation and pain management. Changing the management model for these patients is crucial to improve postoperative cognitive outcomes (O’Brien et al. 2017). The roles of anaesthesiologists are crucial for multidisciplinary perioperative care teams to guide recommendations. The primary responsibilities of multidisciplinary teams are preoperatively educating patients, screening for CI and delirium, implementing non-pharmacological treatments, managing pain, and avoiding antipsychotic medications (Peden et al. 2021). A diverse and interdisciplinary preventive approach can decrease the frequency and duration of POD among older patients undergoing elective (excluding cardiac) surgery and improve their treatment results and prognosis.

The main barriers to MDT implementation are resource limitations and poor interdisciplinary communication, which can be addressed by establishing dedicated perioperative geriatric care teams and regular interdisciplinary meetings. Despite POD being a postoperative complication in older patients, the depths of knowledge among anaesthesiologists regarding delirium guidelines vary, highlighting key barriers to identifying and preventing POD (Ragheb et al. 2023) (Table 5).

**Table 5:**
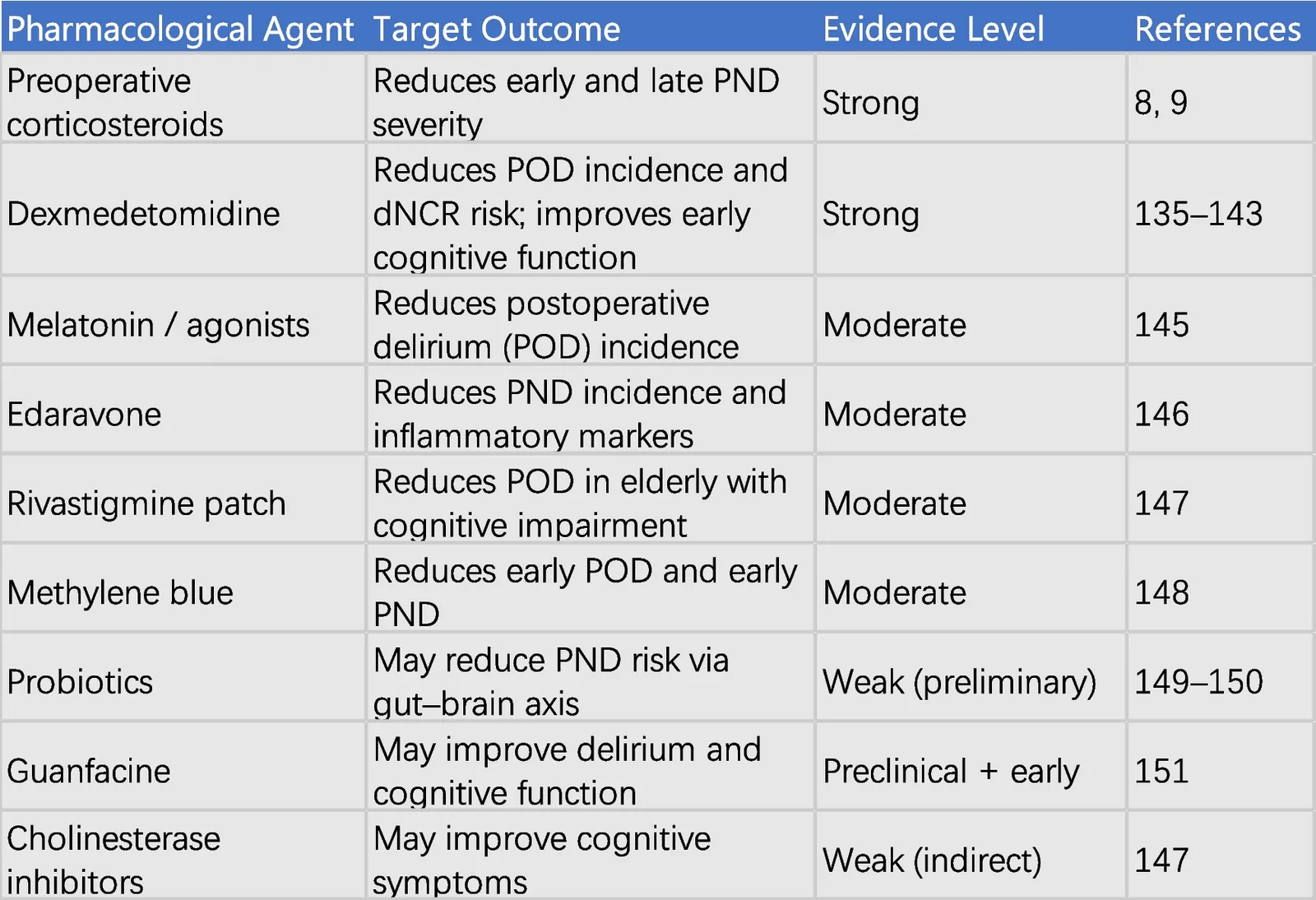
Pharmacological agents for perioperative neurocognitive disorders

## Summary and prospects

Perioperative neurocognitive disorders (PNDs) remain a leading geriatric perioperative complication linked to prolonged hospital stay, increased medical costs, and diminished long-term quality of life. In this updated 2026 narrative review, we systematically synthesized contemporary evidence regarding the pathophysiology, risk stratification, screening tools, and evidence-based interventions for PNDs in older surgical patients, with strict adherence to the 2018 international consensus nomenclature to eliminate terminological confusion.

In terms of risk factors, both modifiable and non-modifiable factors have been relatively confirmed. Advanced age, preoperative cognitive impairment or frailty, persistent intraoperative hypotension (MAP ≤ 65 mmHg for more than 5 min), cardiopulmonary bypass in cardiac surgery, perioperative hypothermia, uncontrolled postoperative pain, sleep disorders, and insufficient multidisciplinary collaboration are all closely associated with the occurrence of PNDs. For clinical risk stratification, bedside screening tools including Mini-Cog, MoCA, MMSE, and FRAIL scale have shown good reliability and can be used in routine clinical practice. In addition, emerging biomarkers such as S100A12, tau-PT217, NfL, and GFAP have shown potential predictive value, but prospective verification is still required before formal clinical application. In terms of intervention strategies, dexmedetomidine has the most sufficient research evidence for the prevention of PNDs. At the same time, non-pharmacological measures such as early postoperative mobilization, multimodal analgesia, sleep improvement, perioperative normothermia maintenance, and multidisciplinary geriatric collaborative management have been recommended as first-line prevention programs in current clinical guidelines.

Although the field of PNDs research has made considerable progress, several key scientific questions remain unresolved. The optimal management thresholds of intraoperative blood pressure, anesthesia depth, and end-tidal CO₂ are still controversial due to the heterogeneity of research results. The long-term effects of different anesthetic drugs and anesthesia methods on cognitive function have not been fully confirmed. Most blood biomarkers lack unified diagnostic thresholds and clear clinical application paths. In addition, whether POD directly leads to long-term cognitive decline or only serves as an external marker of preoperative basic cognitive vulnerability has not been clarified. The preventive effects of adjuvant drugs such as melatonin, probiotics, and cholinesterase inhibitors are only supported by preliminary research evidence, and more large-sample randomized controlled trials are needed for verification.

This review also updates clinical practice by distinguishing strongly supported interventions from weakly evidenced or experimental strategies, helping clinicians avoid overinterpretation of preliminary data. Future research should focus on carrying out standardized multicenter randomized controlled trials to determine the optimal perioperative hemodynamic, anesthetic, and respiratory management goals for brain protection. It is necessary to establish a comprehensive risk prediction model combining demographic data, clinical information, biomarkers, and intraoperative monitoring indicators to achieve personalized risk assessment. Mechanism studies targeting neuroinflammation and gut microbiota regulation are expected to provide new targets for the prevention and treatment of PNDs. Translational research should pay more attention to integrating the latest research results into a clinically operable perioperative brain protection program through multidisciplinary cooperation. At the same time, long-term follow-up cohort studies lasting more than one year after surgery are needed to clarify the long-term cognitive and functional impacts of PNDs.

In clinical practice, anesthesiologists, surgeons, geriatricians, and nursing staff should jointly implement a multimodal and patient-centered management strategy, including preoperative standardized risk screening, individualized intraoperative monitoring and management, and refined postoperative supportive care. The implementation of this integrated management model is expected to effectively reduce the incidence of PNDs, improve perioperative safety, and ultimately protect the long-term cognitive function and quality of life of the increasing number of elderly surgical patients.

## Data Availability

No datasets were generated or analysed during the current study.
